# A Sulfated Polysaccharide from *Saccharina japonica* Suppresses LPS-Induced Inflammation Both in a Macrophage Cell Model via Blocking MAPK/NF-κB Signal Pathways In Vitro and a Zebrafish Model of Embryos and Larvae In Vivo

**DOI:** 10.3390/md18120593

**Published:** 2020-11-26

**Authors:** Shengnan Wang, Liying Ni, Xiaoting Fu, Delin Duan, Jiachao Xu, Xin Gao

**Affiliations:** 1 College of Food Science & Engineering, Ocean University of China, 5th Yushan Road, Qingdao 266003, China; wsn@stu.ouc.edu.cn (S.W.); niliying@stu.ouc.edu.cn (L.N.); xujia@ouc.edu.cn (J.X.); xingao@ouc.edu.cn (X.G.); 2 State Key Lab of Seaweed Bioactive Substances, 1th Daxueyuan Road, Qingdao 266400, China; dlduan@qdio.ac.cn; 3 Key Laboratory of Experimental Marine Biology, Institute of Oceanology, Chinese Academy of Sciences, 7th Nanhai Road, Qingdao 266071, China

**Keywords:** *Saccharina japonica*, polysaccharide, anti-inflammatory, RAW 264.7 macrophages, inflammatory cytokines, zebrafish

## Abstract

Inflammation is a complicated host-protective response to stimuli and toxic conditions, and is considered as a double-edged sword. A sulfated *Saccharina* *japonica* polysaccharide (LJPS) with a sulfate content of 9.07% showed significant inhibitory effects against lipopolysaccharide (LPS)-induced inflammation in RAW 264.7 macrophage cells and zebrafish. Its chemical and structural properties were investigated via HPLC, GC, FTIR, and NMR spectroscopy. In vitro experiments demonstrated that LJPS significantly inhibited the generation of nitric oxide (NO) and prostaglandin E_2_ (PGE_2_) via the downregulation of inducible nitric oxide synthase (iNOS) and cyclooxygenase-2 (COX-2) expression and suppressed pro-inflammatory cytokines tumor necrosis factor (TNF)-α and interleukin (IL)-1β production via the nuclear factor-kappa B (NF-κB) and mitogen-activated protein kinase (MAPK) signal pathways in LPS-induced RAW 264.7 cells. Moreover, LJPS showed strong protective effects against LPS-induced inflammatory responses in zebrafish, increasing the survival rate, reducing the heart rate and yolk sac edema size, and inhibiting cell death and the production of intracellular reactive oxygen species (ROS) and NO. Its convenience for large-scale production and significant anti-inflammatory activity indicated the potential application of LJPS in functional foods, cosmetics, and pharmaceutical industries.

## 1. Introduction

Among the natural products synthesized by plants, polysaccharides are the most abundant components and are widely found in terrestrial plants and seaweed [1]. Polysaccharides from brown algae can be grouped in three principal categories, alginate, fucoidan, and laminarin, which have been investigated extensively over the past few decades [2,3]. They have exhibited various functional properties, such as antibacterial [4], anti-obesity [5], anticoagulant [6], antiviral [7], immunomodulatory [8], and antioxidant [9] activities.

*Saccharina japonica* (*S. japonica*), which is one of the most important and abundant marine resources, is a popular edible brown seaweed cultured in Asian states, especially in China. As one of the most economically important species in marine aquaculture, *S. japonica* was documented as a traditional Chinese medicine 1000 years ago [10]. Many studies have reported various biological activities in polysaccharides isolated from *S. japonica* [8,10,11,12,13,14,15,16,17], but little research has reported their anti-inflammatory activities [18,19].

Inflammation is a protective biological response, usually caused by a range of toxic or stimulus conditions, such as noxious stimuli and cell damage. Correctly regulated inflammatory responses are conducive to physical resistance to the disease, but extended chronic inflammation can cause related autoimmune ailments, such as diabetes, arthritis, asthma, and neurodegenerative diseases [20,21].

During inflammatory responses, macrophage cells secrete cytokines such as tumor necrosis factor (TNF)-α and interleukin, related to multifarious signal pathways. The generation of nitric oxide (NO) and the production of other inflammatory biomarkers such as prostaglandin E_2_ (PGE_2_) are promoted [22,23,24]. Lipopolysaccharide (LPS)-induced inflammation is widely used in an array of assays to study the interference with inflammatory pathways [25]. LPS-stimulated macrophages have been commonly used to evaluate the anti-inflammatory capacities of various natural products [26,27,28]. Moreover, the anti-inflammatory abilities of polysaccharides from *Undaria pinnatifida*, *Ecklonia cava*, *Padina boryana* (formerly *Padina commersonii*), and *Turbinaria ornata* have been investigated using LPS-stimulated RAW 264.7 macrophages [29,30,31,32].

Zebrafish (*Danio rerio*) has generally been recognized as a powerful vertebrate animal model for studies on human diseases [33,34]. There are numerous advantages of using zebrafish, including its highly-developed immune system, its physiological and morphological similarity to mammals, the low maintenance cost, and especially the optical transparency of their embryos and larvae, which facilitates in vivo morphological monitoring [35].

Current anti-inflammatory drugs such as glucocorticoids (GCs) and non-steroidal anti-inflammatory drugs (NSAIDs) are usually suitable for acute inflammation, not exactly for chronic inflammation or related ailments. They usually have ineluctable side effects and an inadequate level of long-term safety [36,37]. Consequently, to explore novel, safer treatment strategies, much attention has been focused on botanical polysaccharides with anti-inflammatory properties, [20]. Two recent studies have reported the anti-inflammatory activities of polysaccharides isolated from *S. japonica.* One regards a purified fucoidan of SF6, which has the potential to inhibit LPS-induced inflammation in macrophages [18], and the other regards a recently purified fucoidan fraction of F4, which shows anti-inflammatory activities both in vitro and in vivo [19]. However, these two polysaccharides were purified fractions. Their industrial application is limited because of the time-consuming extraction and purification process, along with its high cost and extremely low yield. In the present study, we discovered that a sulfated *Saccharina japonica* polysaccharide (LJPS) without chromatographic purification showed significant anti-inflammatory activities. In additional to its low-cost processing, it showed no toxicity at a higher dose and possessed superior anti-inflammatory activities than those of the purified fractions. Because of the potential for large-scale processing and application, its anti-inflammatory activities and mechanisms both in vitro and in vivo in RAW 264.7 cells and zebrafish models were investigated in this study.

## 2. Results

### 2.1. Yield and Physicochemical Properties of LJPS

As shown in Table 1, the LJPS yield was 7.96% according to the dried weight of the seaweed. The total sugar content was 64.42%, determined by the phenol-sulfuric acid method, and the sulfate content was 9.07%. In addition, the content of phenolic compounds and protein were 0.05% and 0.72%, respectively. LJPS possessed a high weight-average molecular weight (M_w_) of 131.5 kDa. The result of monosaccharide components in Table 1 shows that it was mainly composed of fucose (47.15%), galactose (23.02%), and mannose (16.88%). It also contained small amounts of rhamnose (1.18%), xylose (5.53%), and glucose (6.23%). In comparison, the monosaccharide composition was different from previously reported polysaccharides isolated from *S. japonica* [8,12,19,38]. Thus, LJPS is a heteropolysaccharide with high sulfate and fucose content.

### 2.2. Structural Characterization of LJPS

#### 2.2.1. Fourier-Transform Infrared Spectroscopy (FTIR)

**Figure 1:**
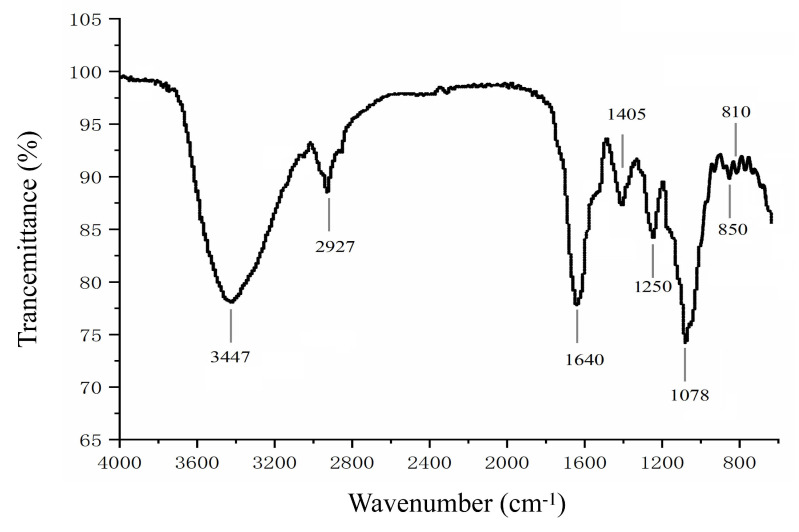
Structural characterization of LJPS by Fourier-Transform Infrared Spectroscopy (FTIR).

The FTIR of LJPS is shown in Figure 1. The strong absorbance peak at 3447 cm^−1^ obviously represents a typical major broad stretching peak of O-H, and the narrow weak peak at 2927 cm^−1^ was assigned to C-H bonds, which is consistent with previous reports [19,39]. Absorption around 1640 cm^−1^ and 1078 cm^−1^ revealed C=O and C-O bonds in the carbohydrate, respectively, which further indicates the presence of a sugar ring structure. In addition, the characteristic peak at 1250 cm^−1^ was caused by an S=O stretching vibration. Absorbance peaks around 810 cm^−1^ and 850 cm^−1^ were associated with a C-O-S stretching vibration. The peak around 850 cm^−1^ suggested sulfate groups located at the axial C4 position [39]. The FTIR results further indicate that LJPS is a sulfated polysaccharide.

#### 2.2.2. NMR Spectroscopy

**Figure 2:**
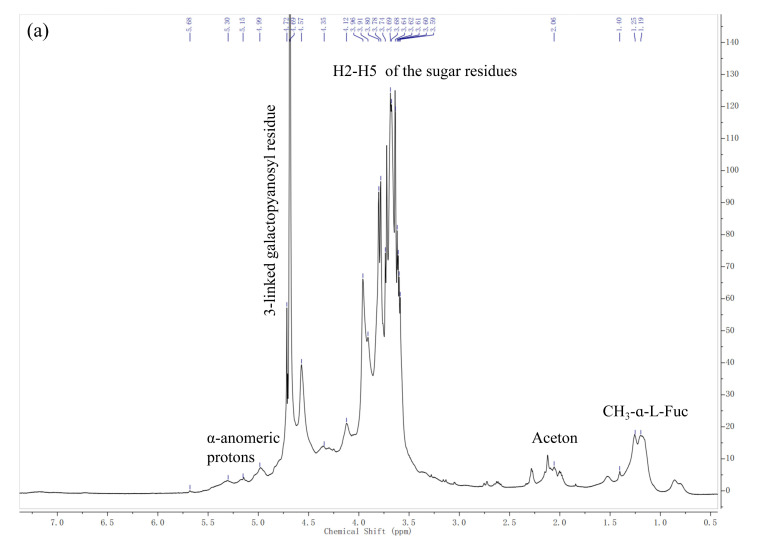

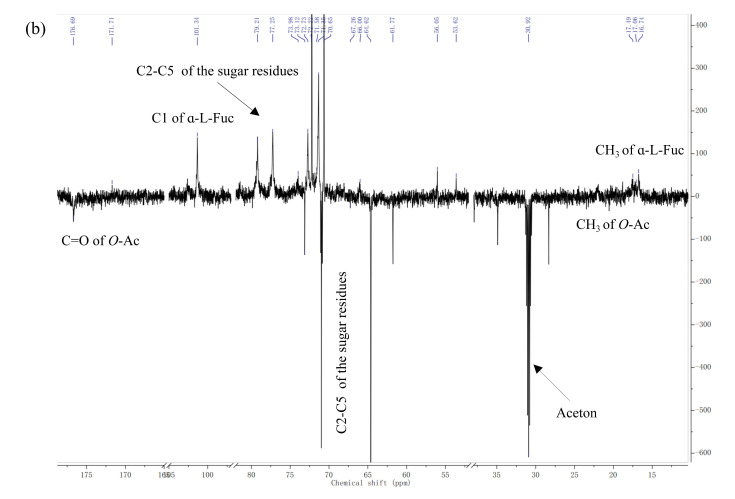
The ^1^H (**a**) and DEPTQ (**b**) spectrum of LJPS.

NMR spectroscopy is a common and powerful tool for the structural characterization of sulfated polysaccharides from seaweed [40]. Like many other fucoidan fractions, LJPS has a quite complicated NMR spectrum. The complexity of the sulfated polysaccharide structure causes most of the ring proton signals at various locations to be concentrated in the narrow range of 3.0–5.0 ppm, and the stacking is serious. Nevertheless, the ^1^H-NMR spectrum can still be analyzed to some extent. The ^1^H-NMR spectrum of LJPS is shown in Figure 2a. The signals at 1.19 ppm represent methyl protons of α-L-fucf-(1→3) residues [19]. The peak at 1.25 and 1.40 ppm was associated with the C6 methyl proton group of L-fucopyranose [19,31,41]. The minor signal region of broadened peaks from 5.0 to 5.7 ppm was associated with anomeric protons of α-L-fucopyranosyl residues [19,41]. The ring protons H2-H5 of the sugar residues were confirmed by the intense unresolved peaks at 3.58–4.71 ppm [19,31]. The intense anomeric signal at 4.57 ppm was associated with H1 of β-galactopyranosyl residues [41]. The signal at 4.69 ppm was assigned to 3-linked D-galactopyranosyl residues [31].

In addition, the structure of LJPS was further analyzed by the ^13^C-DEPTQ spectrum (Figure 2b). The ^13^C-DEPTQ spectrum yields signal and multiplicity information for all carbon types with marked increasing signals of sensitivity-limiting quaternary carbons. In the ^13^C-DEPTQ spectrum of LJPS, several intense signals in the anomeric (100.9–102.6) and high-field (16.7–17.6) regions that are typical of α-L-fucopyranose residues were detected [19]. Vishchuk et al. [38] and Ni et al. [19] ascribed signals at 16.7–17.6 ppm and 100.9–102.6 ppm to C6 and anomeric C1 carbons of α-L-fucopyranose, respectively. The intense signals at 101.3 ppm were attributed to C1 of 1,3-linked α-L-fucopyranose [19]. Intense signals in the 62–82 ppm region were associated with C2-C5 carbons of the pyranoid ring [41]. According to Wang et al. [42], the absence of visible signals around 83.0–86.0 ppm indicated an absence of C2-C4 linked galactose units, which suggested that galactose residues were C1 and C6 that are linked in LJPS. The resonance at 61.8 ppm and 66.1 ppm were assigned to non-6 linked (CH_2_OH) and O6 substituted (CH_2_OR) β-D-galactopyranose residues, respectively [38]. Relative weak carbonyl carbon signals at 171–177 ppm and corresponding methyl carbon signals at 20.5–21.9 ppm were associated with *O*-acetyl groups [38,41].

In general, the combined results indicate that LJPS is a sulfated and partially acetylated galactofucan, the sulfate groups of which are mainly located at the C4 positions of fucopyranose residues. In addition, the main chain of LJPS is composed of (1→3)-α-L-fucose residues, with abundant branches consisting of α-L-fucose and β-D-galactose residues.

### 2.3. Anti-Inflammatory Effects of LJPS In Vitro by LPS-Induced RAW 264.7 Cells

#### 2.3.1. Effect of LJPS on Cell Viability and NO Production

**Figure 3:**
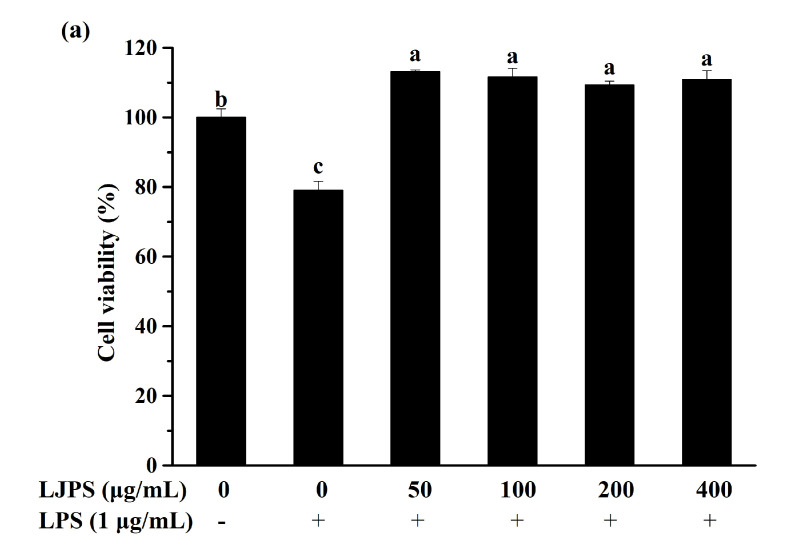

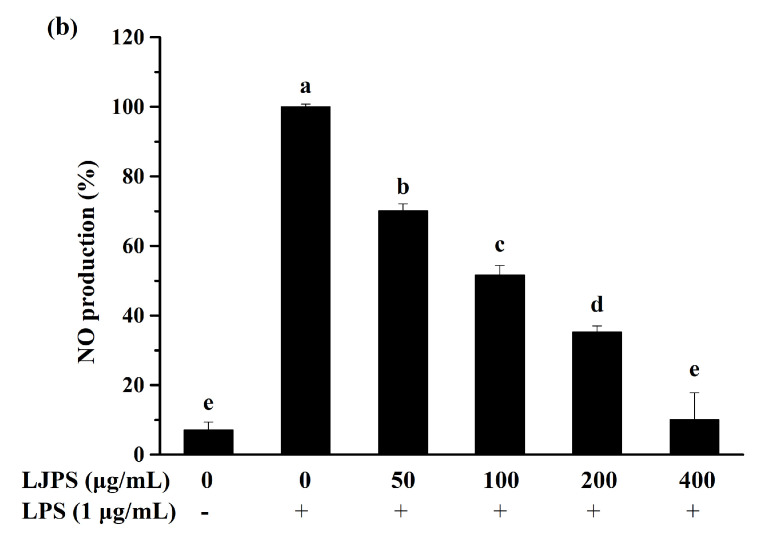
Effects of LJPS on cell viability and NO production in RAW 264.7 macrophages. (**a**) Cell viability; (**b**) NO production. Cell viability was measured by the MTT assay in order to evaluate the cytotoxicity of LJPS in RAW 264.7 cells, and cells treated with LPS were used as the positive control. The levels of NO in LPS-induced RAW 264.7 cells in the presence of LJPS or not were measured by Griess assay. All results are represented as the means ± SD of three independent experiments. Different superscript letters indicate significant differences from each other with *p* < 0.05 (Tukey’s multiple-range test).

Before evaluating the anti-inflammatory effects of LJPS in vitro, a cytotoxicity assay was necessary for estimating the negative effects of LJPS on RAW 264.7 cells. As shown in Figure 3a, the viabilities of RAW 264.7 cells were more than 90% in all experimental groups and even higher than the control group, while the cell viability in the positive control group treated with LPS was decreased to 79.00% compared to those not treated with LPS. LJPS has no obvious cytotoxic effect on RAW 264.7 cells within the concentration range of 50–400 μg/mL. Four experimental groups showed no significant differences from each other via Tukey’s multiple-range test (*p* < 0.05).

To preliminarily investigate the anti-inflammatory effects on LPS-induced RAW 264.7 cells, the production of NO was evaluated by determining the nitrite levels in the medium using a sodium nitrite standard curve [16]. An approximately 14-fold increase in NO production was noted in the LPS-treated group, while LJPS treatment obviously inhibited the production of NO in a dose-dependent manner. Furthermore, the NO production level was 89.98% inhibited by the highest test concentration (Figure 3b).

#### 2.3.2. Effect of LJPS on PGE_2_ and Pro-Inflammatory Cytokines Secretion

**Figure 4:**
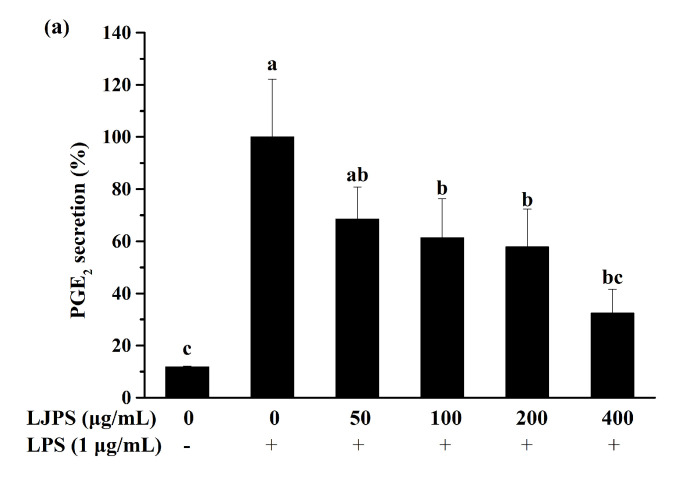

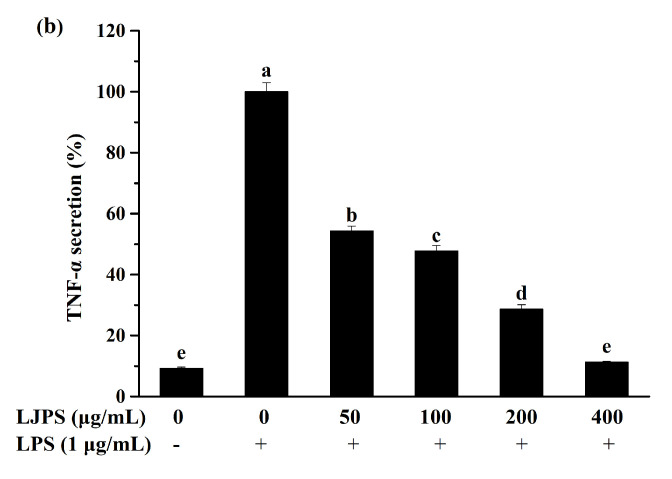

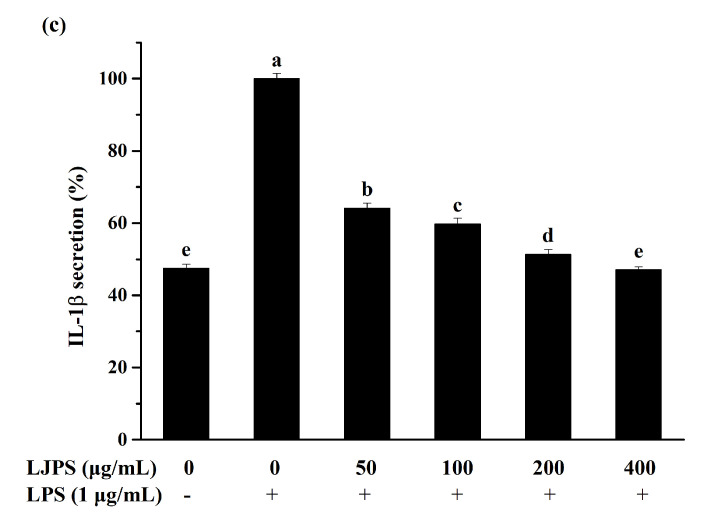
Effect of LJPS on PGE_2_ and pro-inflammatory cytokine secretion in lipopolysaccharide (LPS)-stimulated RAW 264.7 macrophages. (**a**) PGE_2_ secretion; (**b**) tumor necrosis factor (TNF)-α secretion; (**c**) IL-1β secretion. RAW 264.7 cells were pretreated with LJPS (50, 100, 200, and 400 μg/mL) first and then induced with LPS (1 μg/mL). After 24 h of incubation, culture media was collected for analysis of PGE_2_, TNF-α, and interleukin (IL)-1β. All results are represented as the means ± SD of three independent experiments. Different superscript letters indicate significant differences from each other with *p* < 0.05 (Tukey’s multiple-range test).

The levels of selected inflammatory mediators secreted by RAW 264.7 cells were analyzed using corresponding enzyme-linked immunosorbent assay (ELISA) kits. As shown in Figure 4, LPS induction significantly (*p* < 0.05) promoted the secretion of PGE_2_, interleukin (IL)-1β, and TNF-α compared to the untreated group. While similar to nitrite, LPS-induced PGE_2_, TNF-α, and IL-1β production decreased significantly (*p* < 0.05) and dose-dependently when the cells were pretreated with LJPS (Figure 4a–c). Furthermore, the highest test concentration showed the best inhibition ratios of 52.96%, 67.52%, and 88.72% for PGE_2_, TNF-α, and IL-1β, respectively.

#### 2.3.3. Effect of LJPS on iNOS and COX-2 Expression

**Figure 5:**
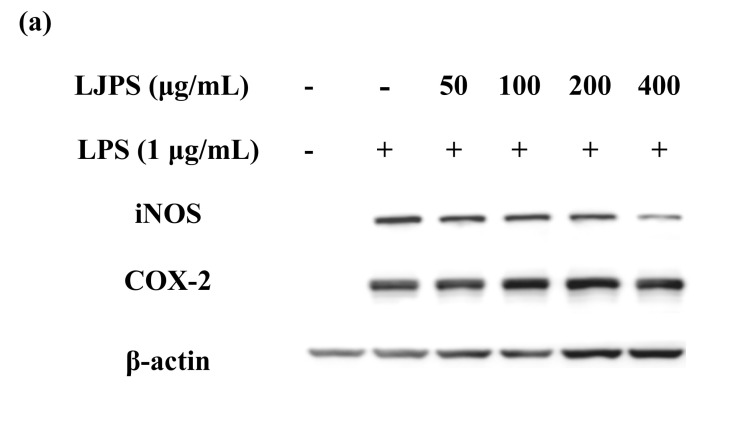

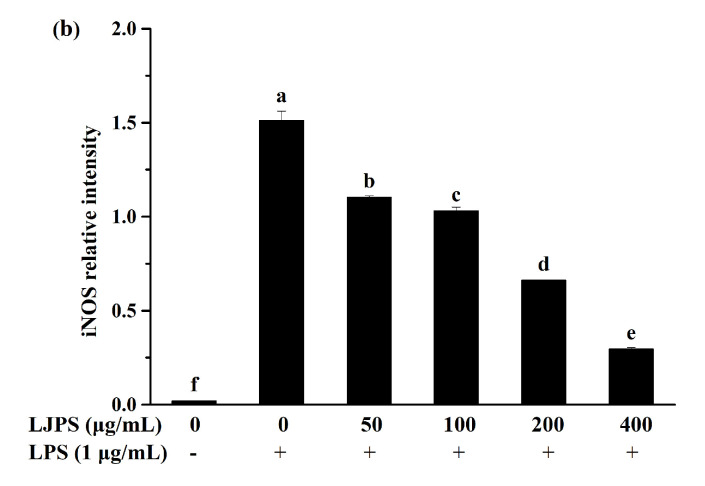

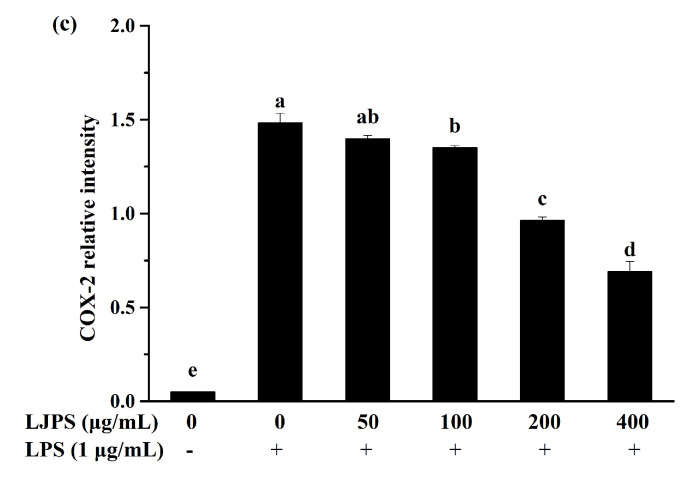
Effect of LJPS on iNOS and COX-2 protein expression in LPS-stimulated RAW 264.7 cells. (**a**) Expression analysis of iNOS and COX-2 evaluated using Western blot; (**b**) iNOS protein expression; (**c**) COX-2 protein expression. Western blot analysis was applied for evaluating iNOS and COX-2 expression at the protein level. The proteins’ levels were determined using Image J software. All results are represented as the means ± SD of three independent experiments. Different superscript letters indicate significant differences from each other with *p* < 0.05 (Tukey’s multiple-range test).

Apart from the levels of NO and inflammatory cytokines, the inducible nitric oxide synthase (iNOS) and cyclooxygenase-2 (COX-2) protein expressions were confirmed as an inflammatory response in macrophages. Protein expressions of iNOS and COX-2 measured by Western blot assay are shown in Figure 5. iNOS and COX-2 expressions in LPS-treated RAW 264.7 cells significantly increased by about 78- and 30-fold compared to the untreated group, respectively (*p* < 0.05). However, the treatment of LJPS at 400 μg/mL decreased iNOS and COX-2 levels to 19.60% and 46.50% of the LPS-induced group, respectively. Thus, the result recommends the pretreatment with LJPS effectively downregulated the LPS-stimulated release of iNOS and COX-2.

#### 2.3.4. Effects of LJPS on Nuclear Factor-Kappa B (NF-κB) and Mitogen-Activated Protein Kinase (MAPK) Signaling Pathway

**Figure 6:**
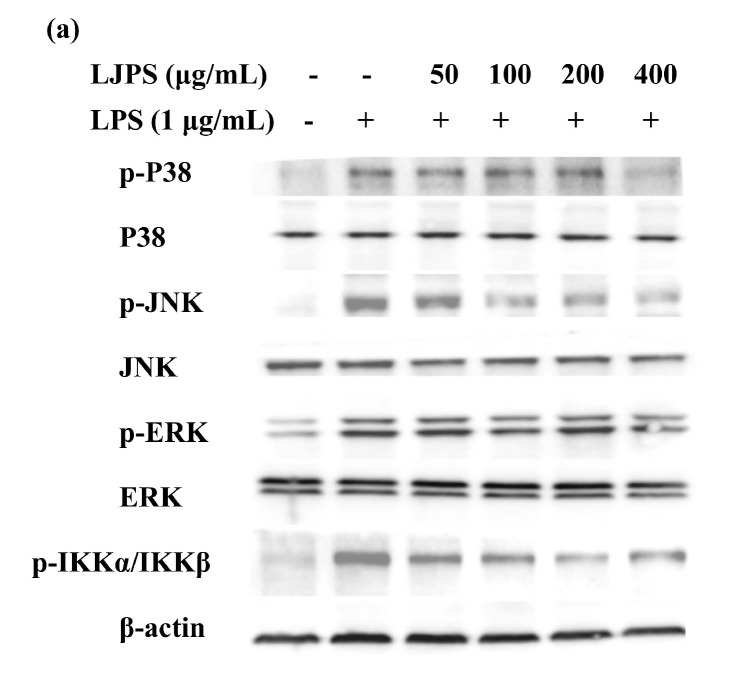

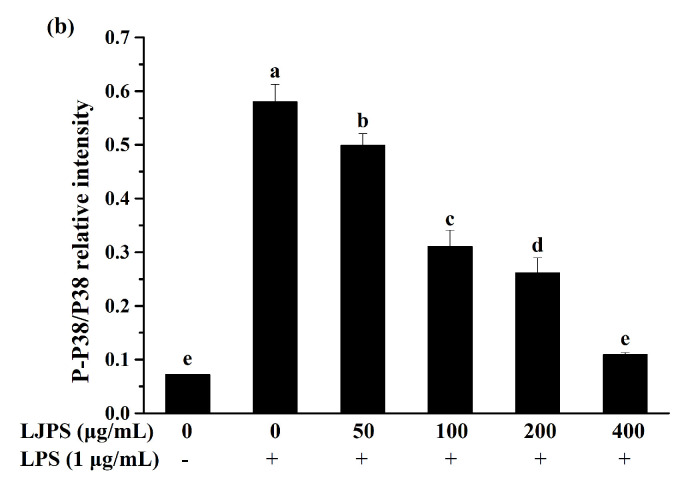

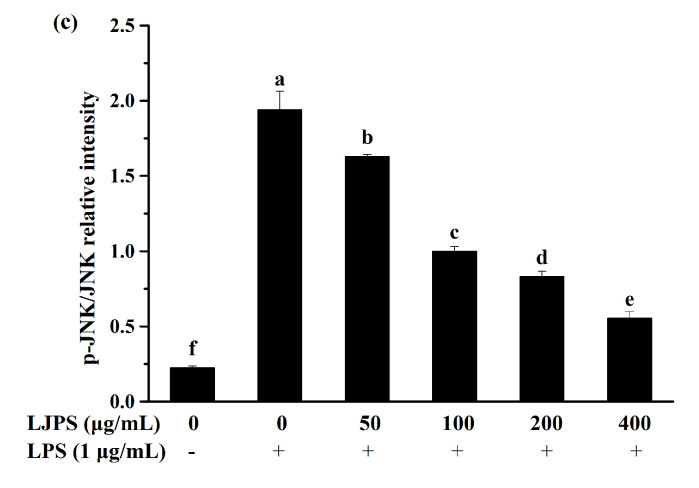

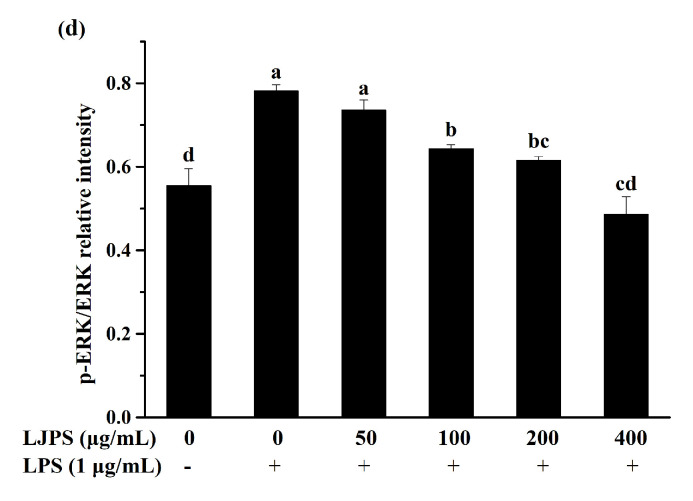

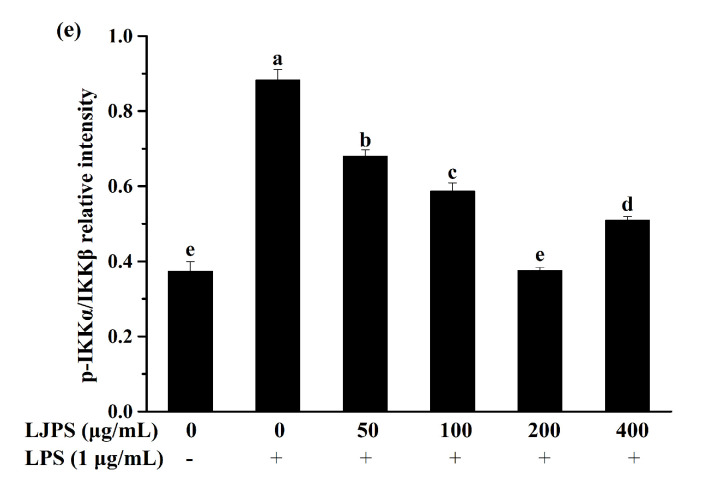
Effect of LJPS on NF-κB and MAPK signaling pathways in LPS-stimulated RAW 264.7 cells. (**a**) Protein expression analysis of NF-κB and MAPK signaling pathways evaluated using Western blot; (**b**) p-P38/P38 relative protein expression; (**c**) p-JNK/JNK relative protein expression; (**d**) p-ERK/ERK relative protein expression; (**e**) p-inhibitor of nuclear factor kappa-B kinase (IKK)α/IKKβ relative protein expression. The relative amounts of each protein were compared with β-actin. All results are represented as the means ± SD of three independent experiments. Different superscript letters indicate significant differences from each other with *p* < 0.05 (Tukey’s multiple-range test).

In the present study, the p38 mitogen-activated protein kinase (p38 MARK), c-Jun N-terminal kinase (JNK), and extracellular signal-regulated kinase (ERK) were analyzed to evaluate the activation of the MAPK signaling pathway. As Figure 6b–d shows, the phosphorylation of the MAPK pathway proteins in the presence of LPS were significantly promoted by about 8.1-, 8.6-, and 1.4-fold, respectively, compared with the control group (*p* < 0.05), while incubation with LJPS significantly inhibited the phosphorylation of MAPK proteins in a dose-dependent manner (*p* < 0.05). At the concentration of 400 μg/mL, p38 MARK, JNK, and ERK phosphorylation levels were respectively suppressed to 18.77%, 28.57%, and 62.15%, compared to the LPS-treated group. Dramatically, 400 μg/mL of LJPS downregulated the phosphorylation of ERK to 0.49 ± 0.04 (relative intensity of p-ERK/ERK), which was even lower than that of the control group, but with no significant difference (*p* < 0.05).

To investigate the activation of the NF-κB pathway and the effects of LJPS on it, related down-stream proteins in the cytosol were analyzed by the Western blot assay. As shown in Figure 6e, LPS stimulation led to activated phosphorylation levels of IKKα/β, which was dampened by pre-treatment with LJPS dose-dependently and significantly. After pre-incubation with different concentrations (50, 100, 200, and 400 μg/mL) of LJPS, compared with the LPS-treated group, the phosphorylation of IKKα/β was downregulated by 22.89%, 33.51%, 57.44%, and 42.31%, respectively. In general, LJPS showed an ability to inhibit the LPS-induced phosphorylation of p38 MARK, JNK, ERK, and IKKα/β in RAW 264.7 cells.

### 2.4. Anti-Inflammatory Effects of LJPS In Vivo by LPS-Induced Zebrafish Model

#### 2.4.1. Effects of LJPS on Heart Rate, Yolk Sac Edema Size, and Survival Rate in LPS-Stimulated Zebrafish Embryos

**Figure 7:**
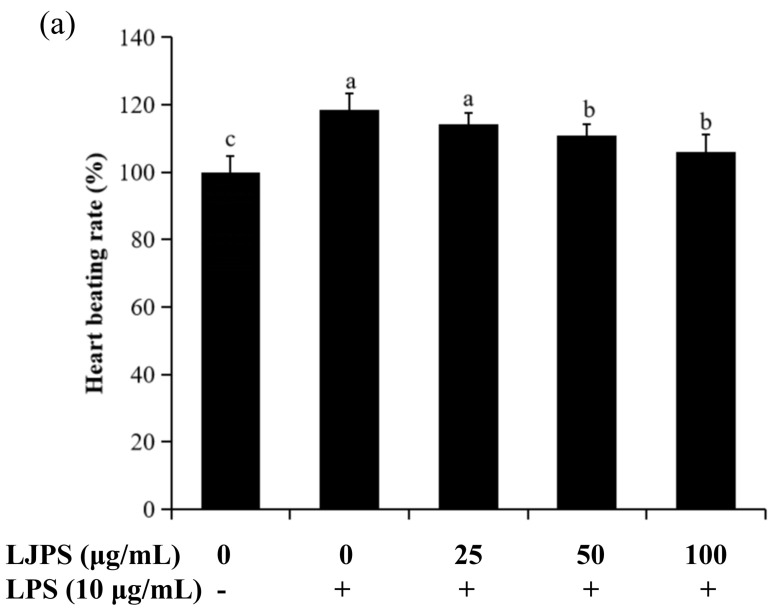

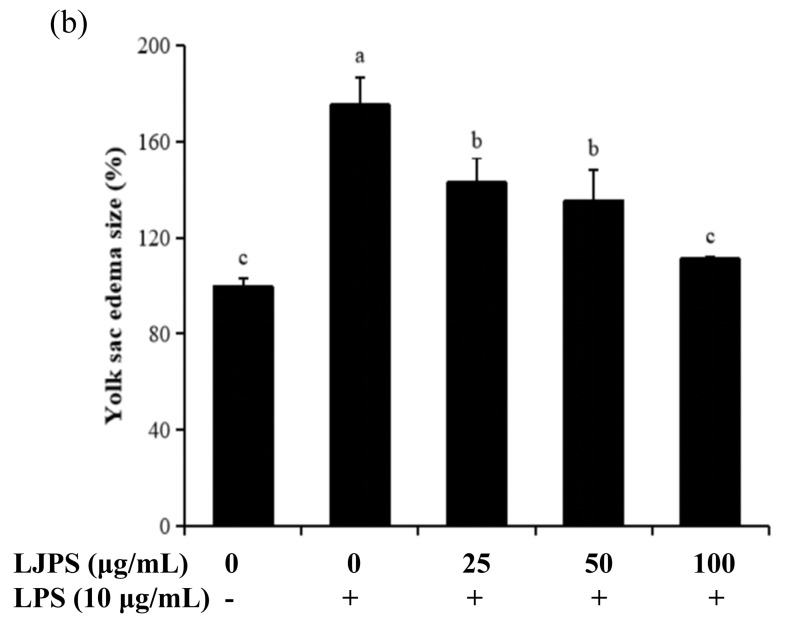

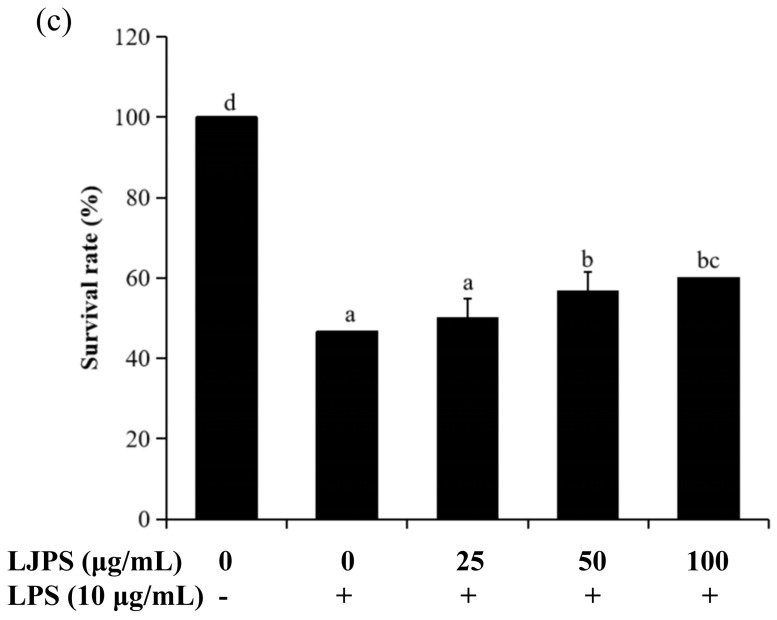
The heart rate, yolk sac edema size, and survival rate of LPS-stimulated zebrafish embryos after pretreatment with LJPS. (**a**) Heart rate; (**b**) yolk sac edema size; (**c**) survival rate. At 30 hpf after LPS (10 μg/mL) treatment, a microscope with a camera and embryos without active avoidance behaviors were used for the measurement of yolk sac edema size. At 2 dpf, heart rate was measured for 1 min in hatched embryos. The survival rate was determined at 3 dpf by counting the surviving hatched embryos. All results are represented as the means ± SD of three independent experiments. Different superscript letters indicate significant differences from each other with *p* < 0.05 (Tukey’s multiple-range test).

Firstly, the toxic effect of LJPS on zebrafish embryos was evaluated by the survival rates (shown in the supplementary data). The results indicate that concentrations of 0–100 μg/mL had no toxic effect, while there was a significant toxic effect on zebrafish embryos when the concentration exceeded 100 μg/mL. Thus, the concentration range of 25–100 μg/mL was chosen for the next part of the anti-inflammatory study in vivo. As shown in Figure 7a, the heart rate of the LPS-treated group was 118.59% compared to the control group (100%), but this decreased to 105.92% after pretreatment with LJPS (100 μg/mL). The differences in the yolk sac edema sizes of zebrafish embryos at 30 hpf are shown in Figure 7b. There were significant decreases in the yolk sac edema sizes of groups that were co-incubated with LJPS when compared to the group only treated with LPS (*p* < 0.05). Figure 7c shows the survival rates of zebrafish embryos induced with LPS or co-incubated with LJPS. The group only treated with LPS decreased to 46.66% compared to the control, but increased to 60.00% after pre-treatment with LJPS (100 μg/mL). Therefore, LJPS could protect zebrafish embryos from phenotypic changes and toxic damage stimulated by LPS.

#### 2.4.2. Effects of LJPS on Cell Death, Reactive Oxygen Species (ROS), and NO Generation in LPS-Stimulated Zebrafish Embryos

**Figure 8:**
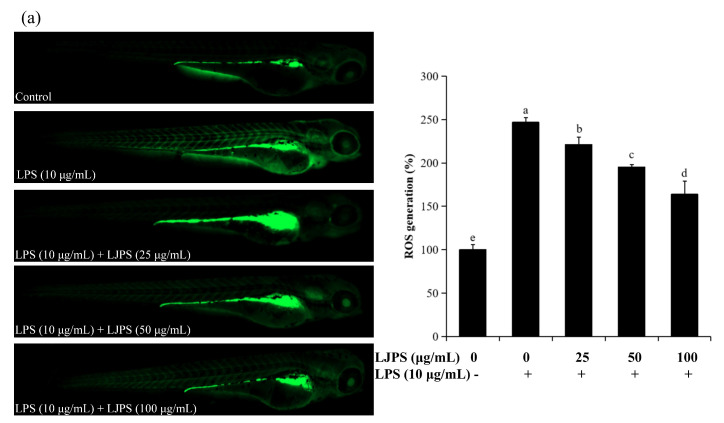

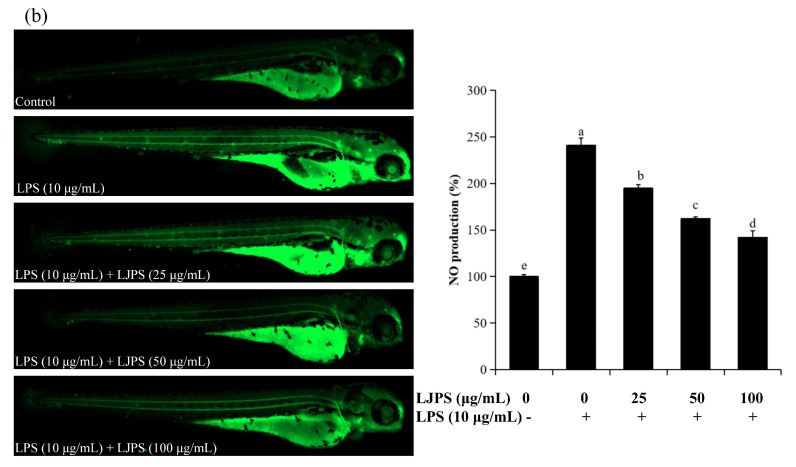

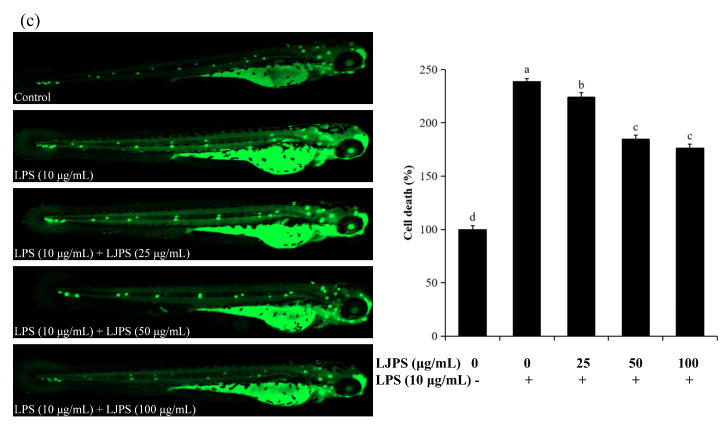
The protective effects of LJPS on cell death as well as ROS and NO production in LPS-stimulated zebrafish embryos. (**a**) ROS production; (**b**) NO production; (**c**) cell death. At 3 dpf after LPS (10 μg/mL) incubation, the levels of cell death and intracellular NO and ROS production were observed and photographed by a fluorescence microscope after staining with acridine orange, diamino fluorophore 4-amino-5-methylamino-2′7′-difluorofluorescein diacetate (DAF-FM DA), and 2, 7-dichlorodihydrofluorescein diacetate (DCFH-DA), respectively. All results are represented as the means ± SD of three independent experiments. Different superscript letters indicate significant differences from each other with *p* < 0.05 (Tukey’s multiple-range test).

The fluorescence images and relative fluorescence intensity analysis of ROS and NO production are shown in Figure 8a,b. Enhanced fluorescence intensities were clearly observed in LPS-treated zebrafish larvae when compared to the control group.

Accordingly, the relative fluorescence intensity analysis suggested that ROS and NO secretion in zebrafish larvae was promoted by treatment with LPS (10 μg/mL), but this decreased remarkably to 163.92% and 142.22%, respectively, in the presence of LJPS (100 μg/mL). In addition, LPS treatment significantly (*p* < 0.05) increased cell death to 238.74% when compared to the untreated group, as shown in Figure 8c. Similarly, after pre-treatment with LJPS, cell death stimulated by LPS was forcefully attenuated in a dose-dependent manner. The result was reduced remarkably to 176.24% at 100 μg/mL compared to the LPS-treated group. The results indicate that LJPS operatively inhibited LPS-induced cell death as well as NO and ROS generation in zebrafish embryos.

## 3. Discussion

In the present study, LJPS with an average molecular weight of 131.50 kDa was isolated from *S. japonica*, with a sulfate content of 9.07% and a high fucose content of 47.15%. Monosaccharide analysis suggested that LJPS contains the most fucose, followed by galactose, which is similar to LJSF4 but different from other reported fucoidan fractions obtained from *S. japonica* [8,16,17,18,19]. The LJPS yield was 7.96%, which was much higher than those of the purified fractions of LJSF4 (2.20%) [19] and SF6 (0.55%) [18] from *S. japonica*. The high yield indicated the great industrial application potential of LJPS. The collection time of the seaweed samples, the extraction methods, and operations during the extraction process might be responsible for the differences in chemical composition and yields. The heterogeneity and complexity of the structure of sulfated polysaccharides, as well as the chemical interactions between each atom, cause difficulty in completely describing its structural characteristics. FTIR and NMR are two powerful tools widely used in the structural characterization of sulfated polysaccharides [40]. According to FTIR and NMR results, LJPS had rather high amounts of α-L-fucopyranose residues, β-d-galactopyranose residues, and *O*-acetyl groups. Thus, LJPS is a partially acetylated, sulfated galactofucan. Past research has indicated that sulfated glycans play an important role in inflammatory responses [20]. Since LJPS contains a high content of sulfate, it may possess strong anti-inflammatory activities.

As a key component of nonspecific immunity, macrophages possess highly malleable phenotypes in huge varieties, which make it play diverse and critical roles throughout most stages of inflammation and disease processes [23]. Thus, RAW 264.7 macrophages were used in our experiment for evaluating in vitro anti-inflammatory abilities. The cytotoxicity of LJPS to macrophages was measured first, and the concentrations of 50, 100, 200, and 400 μg/mL showed no cytotoxicity to macrophages (Figure 3). On the contrary, the cell viabilities of these groups were all more than 100%, which indicated that they have the potential to promote the proliferation of RAW 264.7 macrophages. Therefore, their anti-inflammatory activities were examined in subsequent experiments.

NO is not only an inflammatory marker but also recognized as a pro-inflammatory mediator, which can induce inflammation as a result of overproduction initiated by various types of harmful stimuli. NO is related to the pathogenesis of multifarious inflammation-related disorders, and its secretion is catalyzed by nitric oxide synthases (NOS), such as iNOS. Thus, NO inhibitors are considered important therapeutic drugs against inflammation [43]. In this study, the overproduction of NO was 89.98% inhibited by LJPS at the highest test concentration, while the production of NO showed no significant inhibition by the purified fucoidan of SF6 [18] and only 41% inhibited by LJSF4 [19]. The result indicates the superior anti-inflammatory activity of LJPS to the previously reported fractions.

PGE_2_, another crucial role as a pro-inflammatory mediator, which is derived from the arachidonic acid catalyzed by certain COX, especially COX-2 [44]. COX-2 is inducible in macrophages in response to stimuli and toxic conditions, and the selective inhibition of COX-2 expression can provide an effective therapeutic strategy for the treatment of inflammatory disorders [45]. Previous research suggests that polysaccharides derived from brown seaweed significantly inhibited the release of NO and PGE_2_ in LPS-induced RAW 264.7 cells [31,32]. Similarly, the results in the present study indicate that LJPS effectively suppressed the production of PGE_2_ and the expression of COX-2. Hwang et al. reported that the water extract of *Sargassum hemiphyllum* exhibited the highest depression (55.56%) at 5 mg/mL, while LJPS in this study showed a similar depression of 53.50% at a much lower concentration (400 μg/mL) [46], thereby confirming the outstanding anti-inflammatory ability of LJPS. Moreover, LJPS exhibited a stronger inhibition of NO production compared to the crude polysaccharide of enzyme-assisted extraction from *Padina boryana* (formerly *Padina commersonii*), another brown seaweed [31]. Thus, LJPS is a potential inhibitor of LPS-induced inflammatory responses.

Cytokines, a kind of polypeptide regulator with a low molecular weight, are secreted by cells for an autocrine or paracrine effect. In inflammatory and immune systems, different cytokines can have similar or even identical functional activity. Additionally, they are known as pro-inflammatory cytokines, such as TNF-α and IL-1β [22]. TNF-α can induce acute inflammatory responses, activate the complement system to clear pathogens indirectly, and accelerate a proapoptotic profile in macrophages. IL-1β can also promote the apoptosis of abnormal cells and inflammatory responses mediated by pro-inflammatory mediators [24]. Moreover, the levels of pro-inflammatory cytokines have been accepted as indicators to evaluate the anti-inflammatory capacities in macrophages. Figure 4 indicates that the up-graduated secretion of IL-1β and TNF-α was progressively inhibited at increasing concentrations of LJPS. At an LJPS concentration of 400 μg/mL, TNF-α and IL-1β were effectively decreased to 11.28% and 47.04%, respectively, which indicated that LJPS had a much better anti-inflammatory capacity than the purified fraction (LJSF4); a depression of TNF-α to 55.47% and a depression of IL-1β to 43.30% at the highest concentration were shown [19].

One possible reason is that the non-purified polysaccharide used in this study had a higher non-cytotoxic concentration, meaning that it could be applied at a higher concentration and possessed a better anti-inflammatory activity than the purified fraction. According to our previous report, the carbohydrate content of LJSF4 (56.55%) was lower than that of LJPS (64.42%), while the sulfate content of LJSF4 (30.72%) was much higher than that of LJPS (9.07%) [19]. Previous research has suggested that the sulfate content of polysaccharides is related to anti-inflammatory activity [9,19], while excessive high sulfate amounts may be cytotoxic, which limits their application.

Additionally, the purified fucoidan fraction (SF6) from *S. japonica* has been shown to inhibit the overproduction of TNF-α to 65% at the highest concentration [18], while LJPS showed a much lower inhibition value of 11.28%, which further indicated the superior anti-inflammatory capacity of LJPS.

NF-κB and MAPK signaling pathways are central to the regulation of inflammatory responses in inflammatory and immune systems. NF-κB, a transcription factor, usually performed as an inactive combined former (p50-p65-IκB) in the cytoplasm of unstimulated cells [47,48]. Inflammatory stimuli or pro-inflammatory cytokines, such as TNF-α or IL-1β, can activate NF-κB, causing the phosphorylation of combined IκB and finally enabling the translocation and nuclear localization of NF-κB. Afterward, the secretion of inflammatory cytokines is initiated, and the generation of the pro-inflammatory cytokines is connected to the expression level of NF-κB [49,50]. MAPK signaling pathways, including p38 MARK, ERK, and JNKs, are other important intracellular signaling cascades related to promoting inflammatory responses. Various transcription factors including NF-κB have reportedly been activated by MAPK signaling pathways. Moreover, the MAPK p38 pathway was reported to upregulate the stability of several inflammatory mediator mRNAs, such as TNF-α and COX-2 [51]. Past studies have extensively reported that polysaccharides isolated from various brown seaweeds exhibited an anti-inflammatory capacity via the inhibition of the activation of NF-κB and MAPK signaling pathways in LPS-stimulated macrophages [29,31,46,52]. In the present study, the effects of LJPS on the initiation of NF-κB and MAPKs were evaluated. The data in Figure 6 suggested that the presence of LJPS inhibited NF-κB activation as well as p38 MAPK, ERK and JNK activities in LPS-induced RAW 264.7 cells, which was responsible for the downregulation of pro-inflammatory cytokines and mediators. Thus, the anti-inflammatory mechanisms of LJPS on LPS-stimulated RAW 264.7 macrophages are closely connected with the inhibition of NF-κB and MAPK signaling pathways.

The in vitro anti-inflammatory results were further validated by an in vivo experiment based on an LPS-stimulated zebrafish model. As one of the most important vertebrate animal models, the zebrafish has been used for different kinds of pharmacological research, owing to its distinct advantages compared to cell models and other animal models [53]. Numerous studies have demonstrated that LPS treatment caused toxic impacts on phenotypic changes in zebrafish embryos, such as yolk sac edema size, tail bending, and heart rate. Furthermore, during the LPS-stimulated inflammatory responses in zebrafish larvae, an increased generation of ROS and NO and abnormal cell death induced by the overproduction of ROS and pro-inflammatory cytokines were also observed [52,54]. Polysaccharides derived from brown algae were reported to inhibit cell death and the overproduction of ROS and NO in the LPS-induced zebrafish model [32,52,54]. Lee et al. reported that a fucoidan fraction extracted from *Ecklonia cava* by an enzymic extraction method played a protective role against the toxicity caused by both LPS treatment and tail-cutting in a zebrafish model, which was appraised by analyzing yolk sac edema size and heart rate [34].

In the present study, survival rate, heart rate, and yolk sac edema size were chosen to evaluate both the toxicity and activity of LJPS in LPS-exposed zebrafish. Figure 7 shows that LPS had significant toxic effects on zebrafish embryos, which is consistent with previous reports [54], while the treatment of LJPS showed remarkably protective effects against the toxicity of LPS. These results indicate that LJPS effectively protects zebrafish embryos against the negative effects of LPS treatment. Furthermore, cell death and the levels of ROS and NO generation were analyzed for a more comprehensive evaluation; Figure 8 shows that LJPS treatment effectively downregulated the overproduction of ROS and NO as well as the cell death rate in a dose-dependent manner. A comparison with the documented data [34] found that fucoidan from *Ecklonia cava* reduced the generation of ROS to 83.61% and that of NO to 87.80% at 100 μg/mL in zebrafish larvae subjected to tail-cutting and LPS treatment. LJPS reduced the generation of ROS to 66.42% and that of NO to 59.01% at 100 μg/mL (Figure 8), which suggested that LJPS exhibits superior anti-inflammatory abilities in vivo.

## 4. Materials and Methods

### 4.1. Chemicals and Reagents

Fetal bovine serum (FBS) and Dulbecco’s modified Eagle’s medium (DMEM) were purchased from Gibco (Rockville, MD, USA). 3-(4,5)-Dimethylthiazol-2-yl)-2,5-diphenyltetrazolium bromide (MTT), LPS, Griess reagents, dimethyl sulfoxide (DMSO), 2, 7-dichlorodihydrofluorescein diacetate (DCFH-DA), acridine orange, and diamino fluorophore 4-amino-5-methylamino-2′7′-difluorofluorescein diacetate (DAF-FM DA) were obtained from Sigma Aldrich (St. Louis, MO, USA). Phosphate-buffered saline (PBS), bovine serum albumin (BSA), and penicillin-streptomycin (P/S) were purchased from Solarbio (Beijing, China). ELISA kits for TNF-α, IL-1β, and PGE_2_ were from Dakewei (Shenzhen, China). Primary antibodies against iNOS, COX-2, p38 MARK, phosphorylated p38 MARK, JNK, phosphorylated JNK, phosphorylated IKKα/IKKβ, ERK, phosphorylated ERK, β-actin, and the secondary antibody were obtained from Cell Signaling Technology Inc. (Boston, MA, USA). All other chemicals and reagents used in these experiments were analytical grade.

### 4.2. Plant Material and Extraction

*S. japonica* (J.E. Areschoug) C.E. Lane, C. Mayes, Druehl & G.W. Saunders, the most important economic brown alga in China, was used in this study [55]. The plant materials were cultured and harvested from Lidao Bay (37°13′ N, 122°34′ E) in Rongcheng, Shandong province, China. Forty strains of *S. japonica* were harvested in May 2018. The samples were sun-dried and stored at −20 °C until use. LJPS was prepared based on our previous study [19]. In brief, after removing lipids and pigments by ethanol, *S. japonica* was extracted with ultrapure water at 120 °C for 2 h, and the sulfated polysaccharide (LJPS) was obtained and purified by adding 2% aqueous CaCl_2_ (*w*/*v*) and ethanol precipitation.

### 4.3. Chemical Analysis of LJPS

Total sugar content was measured by the phenol-sulfuric acid method using the fucose standard curve as described by DuBois et al. [56]. The sulfate content of LJPS was detected according to a previously reported method [57]. The total phenol content was determined by a colorimetric method with the Folin-Ciocalteu reagent [58]. The protein percentage was quantified by the Folin-phenol assay, with BSA as the standard [59].

### 4.4. Structural Characterization of LJPS

#### 4.4.1. Analysis of Monosaccharide Composition

Monosaccharide composition analysis was carried out via a gas chromatographic system as reported by Zha et al. [17]. In brief, LJPS was reduced with NaBH_4_ and acetylated with acetic anhydride-pyridine (1:1, *v*/*v*) after it was completely hydrolyzed in trifluoroacetic acid (TFA, 2.0 M). The acetylated aldononitrile derivatives were then detected by a 6890 N gas chromatographic system (Agilent 6890 N, Agilent, Palo Alto, CA, USA).

#### 4.4.2. Determination of M_W_

The molecular weight analysis of LJPS was conducted using HPSEC-MALLS-RID according to our previous report [19]. The experiment was performed on a multiangle laser light scattering detector (MALLS, DAWN HELEOS Ⅱ, Wyatt Technology Co., Santa Barbara, CA, USA) with a refractive index detector (RID) in an Agilent 1260 HPLC system (Agilent Technologies, Palo Alto, CA, USA) coupled with TSK gel GMPWXL (300 mm × 7.8 mm, TosoHaas Corp., Tosoh, Japan) columns in series. A 0.1 M aqueous solution of Na_2_SO_4_ was used as the mobile phase. The molecular weight was calculated by Astra software (Version 6.0.2, Wyatt Tech. Corp., Santa Barbara, CA, USA), according to the standard curve of dextran.

#### 4.4.3. Analysis of Fourier-Transform Infrared Spectroscopy (FTIR)

The samples for the FTIR analysis were prepared by homogenizing with KBr powder and then pressing into 1 mm pellets. The FTIR spectrum from 400 to 4000 cm^−1^ were captured on a spectrometer (Nicolet 6700; Thermo Scientific, Waltham, MA, USA).

#### 4.4.4. Analysis of NMR Spectroscopy

The NMR spectrum (^1^H and DEPTQ) of LJPS was obtained based on our previous study [19]. The samples (100 mg) were dissolved in D_2_O (950 µL) containing 50 µL of deuterated acetone. A Bruker Avance III 600 MHz NMR spectrometer (Bruker, Rheinstetten, Germany) was used for the experiment.

### 4.5. In Vitro Cell Experiments

#### 4.5.1. Cell Line and Culture

RAW 264.7 macrophage cells (MFN048) were purchased from FuDan IBS Cell Center (Shanghai, China). The cells were maintained in DMEM supplemented with 1% P/S and 10% FBS and at 37 °C in a 5% CO_2_ humidified incubator (HERAcell 150i, Thermo, Waltham, MA, USA).

#### 4.5.2. Cell Viability Measurement Using the MTT Assay

RAW 264.7 macrophage cells were seeded into a 96-well plate at a density of 1.5 × 10^5^ cells/well and incubated for 24 h. Cell viability was determined after incubation for another 24 h with newly prepared LJPS (50, 100, 200, and 400 μg/mL in DMEM). MTT solution (2 mg/mL in DMEM) was added to every well after removing the medium. Finally, the supernatants were removed, and 150 μL of DMSO was added to every well prior to absorbance (PowerWave XS, BioTek, VT, USA) measurements at 490 nm [60].

#### 4.5.3. Measurement of NO Production, PGE_2_ and Cytokines Secretion

Exponentially growing cells (1.5 × 10^5^ cells/well) were seeded and incubated for 24 h, and subsequently treated with LJPS (50, 100, 200, and 400 μg/mL in DMEM) for 1 h, after which another 24 h of treatment with LPS (1 μg/mL in DMEM) was given. The conditioned culture media were collected for analyses of NO and cytokine production. NO generation was determined by the Griess assay [8]. The amounts of secreted cytokines (TNF-α, IL-1β, and PGE_2_) were measured using ELISA kits [54]. The cells only treated with LPS were used as a positive control, whereas untreated cells were used as normal control.

#### 4.5.4. Western Blot Analysis

Western blot analysis was used for checking the results of ELISA further. RAW 264.7 cells (1.5 × 10^5^ cells/well) were seeded into 6-well plates and treated as mentioned above. The cells were collected in lysis buffer after washing three times with PBS. Afterwards, the lysates were clarified, and the supernatant fraction was collected. The experiment was then carried out as described preciously by Ni et al. [19].

### 4.6. In Vivo Zebrafish Analysis

#### 4.6.1. Origin and Maintenance of Zebrafish

Adult zebrafish were obtained from the Laboratory of Molecular Medicine (Ocean University of China, Qingdao, China) and subsequently maintained and interbred according to the previous study [54]. The automatic circulation system (ESEN, Beijing, China) was used for this experiment. This experiment was approved by the ethical committee of experimental animal care at the College of Food Science and Engineering at the Ocean University of China (Approval No. 2019-05).

#### 4.6.2. Application of LPS and/or LJPS to Zebrafish Embryos

Sexually mature zebrafish were induced to spawn in the morning by turning on the light, after which the fertilized embryos were collected within 30 min. Embryonic development stages were monitored continuously. Normotrophic contemporized embryos were selected and transferred to 12-well plates (15 embryos/well) containing embryo media before incubating with LJPS (25, 50, 75, and 100 μg/mL in the embryo medium) from 8 h post-fertilization (hpf). After incubation for 1 h, LPS (10 μg/mL) was applied to induce inflammation, except the control. At 24 hpf, the medium was replaced by an equal volume of fresh embryo media and then replaced every 24 h. The embryos only treated with LPS were used as the positive control, whereas untreated embryos were used as normal control.

All incubation was given at 28.5 ± 1 °C in an incubator.

#### 4.6.3. Determination of Heart Rate, Yolk Sac Edema Size, and Survival Rate

Survival rate was determined 3 days post-fertilization (dpf) by counting the hatched embryos that survived [54]. The yolk sac edema size and heart rate were measured according to a protocol reported by Lee et al. [34]. At 30 hpf after LPS treatment, a microscope (A1R HD25, Nikon, Tokyo, Japan) with a camera was used for the analysis of the yolk sac edema size [47]. Heart rate was measured for 1 min and recorded at 2 dpf with the help of the microscope. Values were expressed as the average heart rate per minute [61].

#### 4.6.4. Production of ROS and NO, Cell Death and Image Analysis

The ROS and NO levels as well as cell death were measured based on the reported methods modified by Zou et al. [54]. In brief, at 3 dpf, the hatched larvae were stained for specified periods with specific fluorescent dyes after they were transferred to 24-well plates. The stained zebrafish larvae were then visualized and photographed after being washed three times and anesthetized. Fluorescent dyes DCFH-DA, DAF-FM DA, and acridine orange were used to measure the generation of ROS and NO and the intracellular cell death, respectively [62,63,64]. The fluorescence intensity of each zebrafish larva was quantified with the Image J program (Version 1.8.0, National Institutes of Health, Bethesda, MD, USA).

### 4.7. Statistical Analysis

All experiments were carried out in independent triplicates, and each experiment had at least three parallel groups. All results were expressed as the mean ± standard error. Significant differences were determined via IBM’s SPSS (Version 19.0, SPSS Inc., Chicago, IL, USA) Statistics by one-way analysis of variance (ANOVA). Values with *p* < 0.05 were significantly different. Different letters indicate a significant difference between the compared pairs of individual means, while the same letter means no significant difference between the compared pairs of individual means.

## 5. Conclusions

This study discloses an inexpensive and effective extraction method to isolate a sulfated polysaccharide from *S. japonica* and showed a relative high yield of 7.96%. The evaluated FTIR and NMR results alongside the monosaccharide composition analysis demonstrated the structural characteristics of LJPS. The in vitro results indicate that LJPS acted against LPS-induced inflammation damage to RAW 264.7 cells through NF-κB and MAPKs signaling pathway inhibition. In addition, anti-inflammatory effects of LJPS were observed in vivo; LJPS was shown to protect zebrafish embryos from both phenotypic changes and LPS-exposed toxicity, and reduce LPS-induced ROS and NO generation as well as cell death. Furthermore, the results suggest that the anti-inflammatory capacities of LJPS are superior to those of other reported purified fucoidan fractions. Effective anti-inflammatory activities, a low toxicity, and an economical processing method with a high yield indicate that LJPS can be feasibly produced on a large scale and applied as an anti-inflammatory agent in the functional food, cosmetic, and pharmaceutical industries.

## Figures and Tables

**Table 1:** Yield, molecular weight, and chemical and monosaccharide composition of LJPS.

	LJPS
Yield (%)	7.96 ± 0.85
Molecular weight (kDa)	131.50
Chemical composition	
Total sugar (%)	64.42 ± 0.59
Sulfate (%)	9.07 ± 0.19
Phenol (%)	0.05 ± 0.01
Protein (%)	0.72 ± 0.06
Monosaccharide composition (%)	
Rhamnose	1.18
Fucose	47.15
Xylose	5.53
Mannose	16.88
Galactose	23.02
Glucose	6.23

## Supplementary Materials

The following are available online: https://www.mdpi.com/1660-3397/18/12/593/s1. Figure S1: Monosaccharide and LJPS composition determined by gas chromatograph; Figure S2: HPSEC profile of the molecular weight distribution of LJPS; Figure S3: toxic effect of LJPS on the survival rate of zebrafish embryos.
