# Supplementary material for: A Sulfated Polysaccharide from Saccharina japonica Suppresses LPS-Induced Inflammation Both in a Macrophage Cell Model via Blocking MAPK/NF-κB Signal Pathways In Vitro and a Zebrafish Model of Embryos and Larvae In Vivo
Source: Mar Drugs. 2020 Nov 26;18(12):593. doi: 10.3390/md18120593 (PMC7760670; doi:10.3390/md18120593)
Supplement: Supplementary file 1 [file marinedrugs-18-00593-s001.pdf]

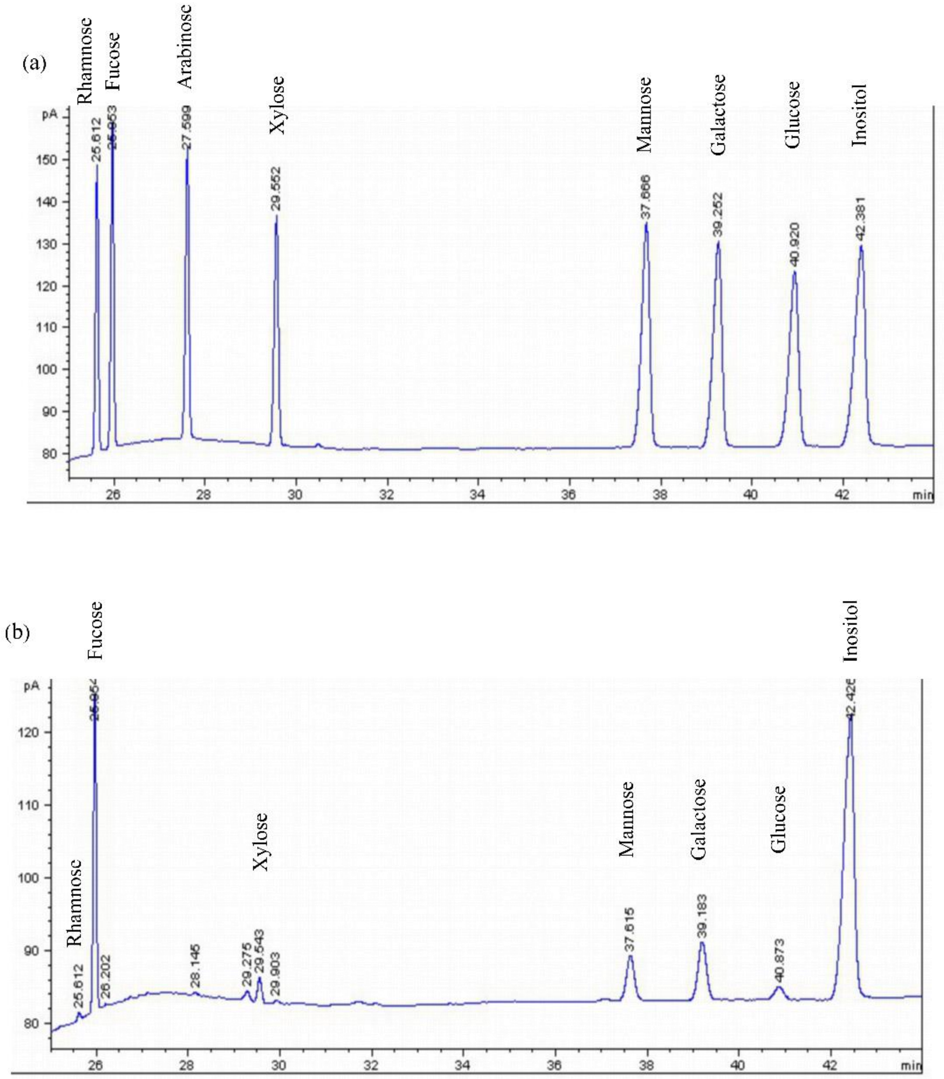

Figure 1. Monosaccharide composition of LJPS determined by gas chromatograph. (a) GC spectrum of acetylated aldononitrile derivatives of seven standard monosaccharides; (b) GC spectrum of acetylated aldononitrile derivatives of LJPS.

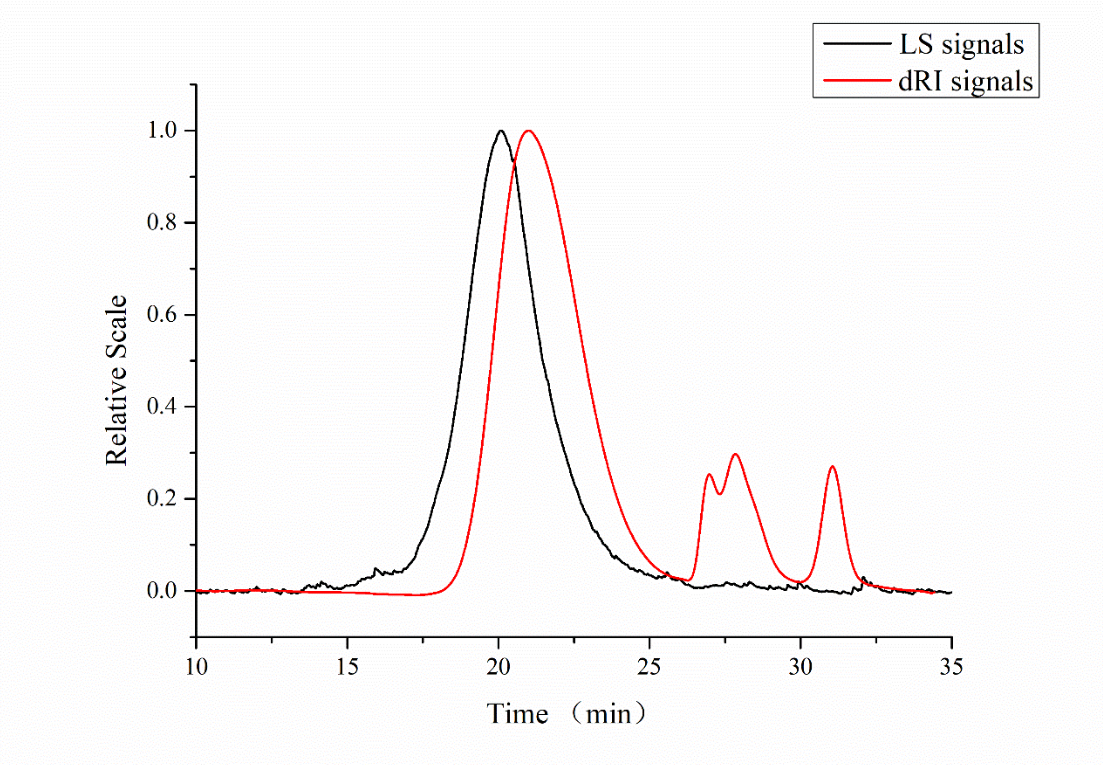

Figure 2. HPSEC profile with the static light scattering and refractive index signals of the molecular weight distribution of LJPS.

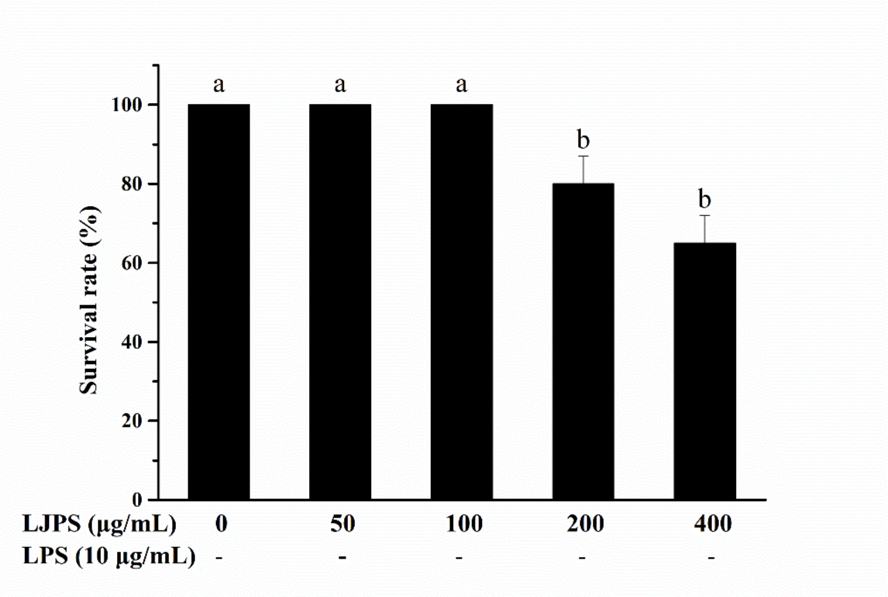

Figure 3. Toxic effect of LJPS on survival rate of zebrafish embryos. After treatment with LJPS, the survival rate was determined at 3 dpf by counting the surviving hatched embryos. All results were represented as means ± SD of three independent experiments. Different superscript letters indicate significant differences from each other with $p < 0.05$ (Turkey' s multiple-range test).
